# Computertomography-Based Prediction of Complete Response Following Neoadjuvant Chemoradiotherapy of Locally Advanced Rectal Cancer

**DOI:** 10.3389/fonc.2021.623144

**Published:** 2021-05-31

**Authors:** Marina Maslova, Heinz Herden, Karin Schork, Michael Turewicz, Martin Eisenacher, Roland Schroers, Alexander Baraniskin, Thomas Mika

**Affiliations:** ^1^ Department of Radiology, Neuroradiology and Nuclear Medicine, Knappschaftskrankenhaus Bochum, Ruhr University Bochum, Bochum, Germany; ^2^ Department of Radiology, VAMED Clinic, Bad Berleburg, Germany; ^3^ Medizinisches Proteom-Center, Ruhr University Bochum, Bochum, Germany; ^4^ Department of Medicine, Hematology and Oncology, Knappschaftskrankenhaus Bochum, Ruhr University Bochum, Bochum, Germany

**Keywords:** locally advanced rectal cancer, neoadjuvant chemoradiotherapy, pathologic complete response, predictive mode, watch and wait, computer tomography

## Abstract

Therapeutic strategies for patients with locally advanced rectal cancer (LARC) who are achieving a pathological complete response (pCR) after neoadjuvant radio-chemotherapy (neoCRT) are being increasingly investigated. Recent trials challenge the current standard therapy of total mesorectal excision (TME). For some patients, the treatment strategy of “watch-and-wait” seems a preferable procedure. The key factor in determining individual treatment strategies following neoCRT is the precise evaluation of the tumor response. Contrast-enhanced computer tomography (ceCT) has proven its ability to discriminate benign and malign lesions in multiple cancers. In this study, we retrospectively analyzed the ceCT based density of LARC in 30 patients, undergoing neoCRT followed by TME. We compared the tumors´ pre- and post-neoCRT density and correlated the results to the amount of residual vital tumor cells in the resected tissue. Overall, the density decreased after neoCRT, with the highest decrease in patients achieving pCR. Densitometry demonstrated a specificity of 88% and sensitivity of 68% in predicting pCR. Thus, we claim that ceCT based densitometry is a useful tool in identifying patients with LARC who may benefit from a “watch-and-wait” strategy and suggest further prospective studies.

## Introduction

Colorectal cancer (CRC) is a common malignancy worldwide, of which 30% of cases develop in the rectum (1, 2). For eligible patients with locally advanced rectal cancer (LARC), neoadjuvant chemoradiotherapy (neoCRT) followed by total mesorectal excision (TME) is the standard treatment to reduce local tumor recurrences and facilitate surgery by tumor size (3, 4). The response of LARC to neoCRT fluctuates broadly, ranging from rare tumor progression to pathological complete response (pCR), with no viable cancer cell residuals in the surgical specimen in up to 33% of patients (3–5).

For patients with absent tumor mass after neoCRT in multiple diagnostic examinations, a “watch-and-wait” strategy, instead of TME, as an individual treatment approach is being increasingly discussed (6–8). Thus, contemporary studies are evaluating intensified primary CRTs, e.g. by addition of chemotherapy agents (oxaliplatin) and prolonged duration of CRT, as a potential definitive and curative treatment (9, 10).

The precise evaluation of tumor responses represents a key factor in determining individual treatment strategies following CRT. Differentiation of post-treatment fibrosis, edema, and residual tumor after CRT in LARC-imaging is one major challenge in implementing rectal preservation strategies.

Currently, different ultrasound techniques, magnetic resonance imaging (MRI), and fluorodeoxyglucose-positron emission tomography (FDG-PET) are widely used for restaging, however, there are still significant limitations for each approach. MRI improves preoperative staging accuracy but has limited sensitivity and specificity to predict pCR (11, 12). Thus, a combination of different methods including MRI, endosonographic ultrasound and digital-rectal examination is currently used for restaging after neoCRT. However, predicting pCR after neoCRT is an object of contemporary research. About half of the patients achieving clinical CR after neoCRT reveal persistence of malignant cells in resected specimens (13), indicating an unmet clinical need of improved staging procedures.

In this study, we focused on CT-densitometry based on Hounsfield units (HU) as assessed by X-ray attenuation. By now, CT-densitometry has repeatedly been reported as an effective imaging technique to differentiate benign from malignant lesions in different cancer types (14–17). Here, we hypothesized that densitometry based on contrast-enhanced CT-scanning (ceCT) has comparable potential to discriminate pCR from patients harboring residual tumor after neoCRT.

In this study, we analyzed HU changes in pre- and post-neoCRT CT-scans in a concordant region of interest (ROI) in rectal tumor areas. In patients with LARC, we were able to demonstrate significant correlations with pCR. To our knowledge, this is the first study showing ceCT-densitometry to predict rectal tumor responses following neoCRT.

## Methods

### Patient Acquisition

Based on ICD codes, patients with LARC treated in our institution between 06/2012 and 04/2020 were identified. The diagnosis of rectal cancer had to be confirmed by histopathological examination. Patients were included if pre- and post-neoCRT contrast ceCT were available and total mesorectal excision was performed after neoCRT. Radiotherapy comprised a total dose of 50.4 Gy. Time from pre-neoCRT diagnostics to the beginning of treatment had to be < 6 weeks. Time from post-neoCRT to surgery had to be <8 weeks. The TNM stage before treatment (iTNM) was set according to routine clinical examination including magnetic resonance imaging (MRI) and endoscopic examination. The post-treatment TNM stage was defined according to the pathologic report (ypTNM). The cancer stage was finally defined according to the American Joint Committee on Cancer (AJCC) with a pathologically confirmed locally advanced rectal adenocarcinoma (T3– T4, any N, M0/any T, N1–N2, M0). A histopathological report with tumor regression grading according to Dworak and a report of the percentage of residual tumor cells had to be available. The ethical committee of the Ruhr-University Bochum approved the study (#20-7013-BR).

### Imaging Techniques

**Figure 1:**
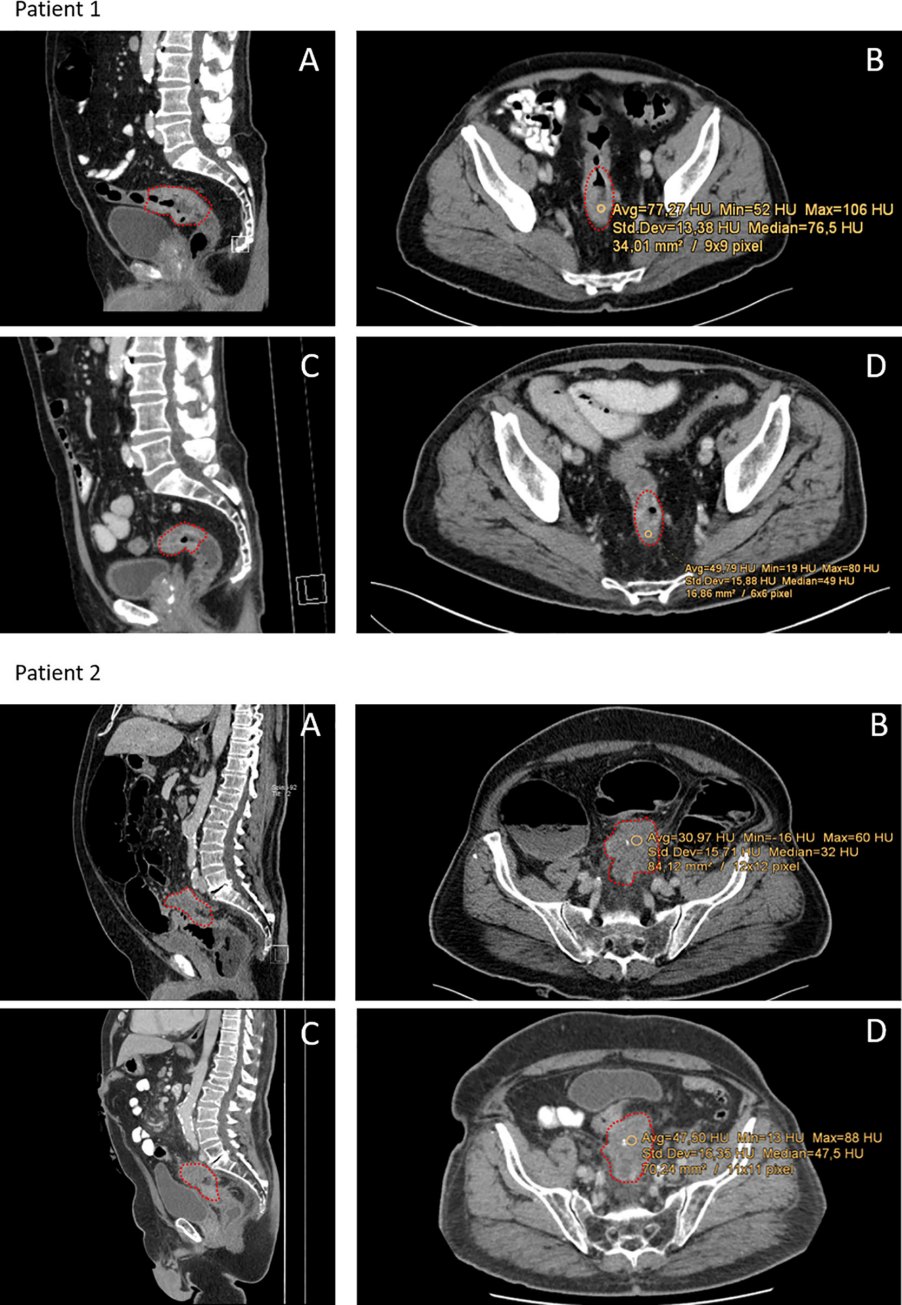
Representative CT-scans of patients with different responses towards neoCRT. Red dots indicate the tumor region. Patient 1: Patient with complete pathological response (regression grade 4 according to Dworak). **(A, B)** Show pre-neoCRT CT-scans in sagittal and axial profile. **(C, D)** Show CT-scans post-neoCRT. Median HU of the tumor was 76.5 HU pre- and 49 HU post-neoCRT. Patient 2: Patient with progressive disease and regression grade 1 according to Dworak and 80% viable tumor cells. **(A, B)** Show pre-neoCRT scans, **(C, D)** post-neoCRT CT-scans. Median HU increase from 31 to 48.

All CT scans were performed in clinical routine settings with Siemens SOMATOM Definition AS (Siemens Healthcare, Forchheim, Germany) set to 40 or 64 slices and Imeron400 contrast agent (Bracco Imaging, Germany). CT settings were the same for all patients analyzed. Images were analyzed in the portal venous phase with 70 s delay after infusion of the contrast agent. Tube voltage was 120kV in both arterial and portal-venouse phase. For detailed imaging settings, please see the **Supplemental Material** (**Table S1**). The tumor’s size was measured by the largest caliber in axial and sagittal plain. The region of interest (ROI) measuring Hounsfield units was set manually in the center of the tumor, avoiding cystic or necrotic regions and not exceeding towards the bowel wall. ROI in post-neoCRT scans were set as close as possible to the pre-neoCRT ROI, guided by bone and organ structures (**Figure 1**). HU were calculated by the formula HU = µ-µ(H_2_O)/µH_2_O (µ:attenuation coefficient). Size of the ROI could differ between pre- and post-neoCRT imaging, with respect to the tumor size. For large or circular tumors, medium values of multiple ROI of the tumor core were used. For very small tumors, not clearly definable in ceCT, MRI was used to identify the tumor region. In this case, the ROI for ceCT based densitometry was set according to concordant MRI images. All images were evaluated by two radiologists with JiveX PACS software (Visus Health IT, Bochum, Germany).

Assessment of response towards neoCRT by MRI and endosonographic ultrasound were extracted from medical reports.

### Statistical Analysis

Percent change of density was calculated as following: (HU post-neoCRT – HU pre-neoCRT)/HU pre-neoCRT *100. Thus, the decline of density was greater in patients with more negative values. Data was analyzed and processed with Graphpad Prism 6 (GraphPad Software, Inc., San Diego, CA). The correlation was analyzed by Pearson correlation and an unpaired t-test. Welch’s t-test was applied to analyze patients with pCR and those with residual tumor cells. A value of p<0.05 was considered as a significant difference in all t-tests applied (*= p<0.05, ** = p<0.01, *** = p<0.001). Specificity, sensitivity, and negative- and positive predictive values were calculated by two-by-two tables.

## Results

### Patient’s Characteristics

**Figure 2:**
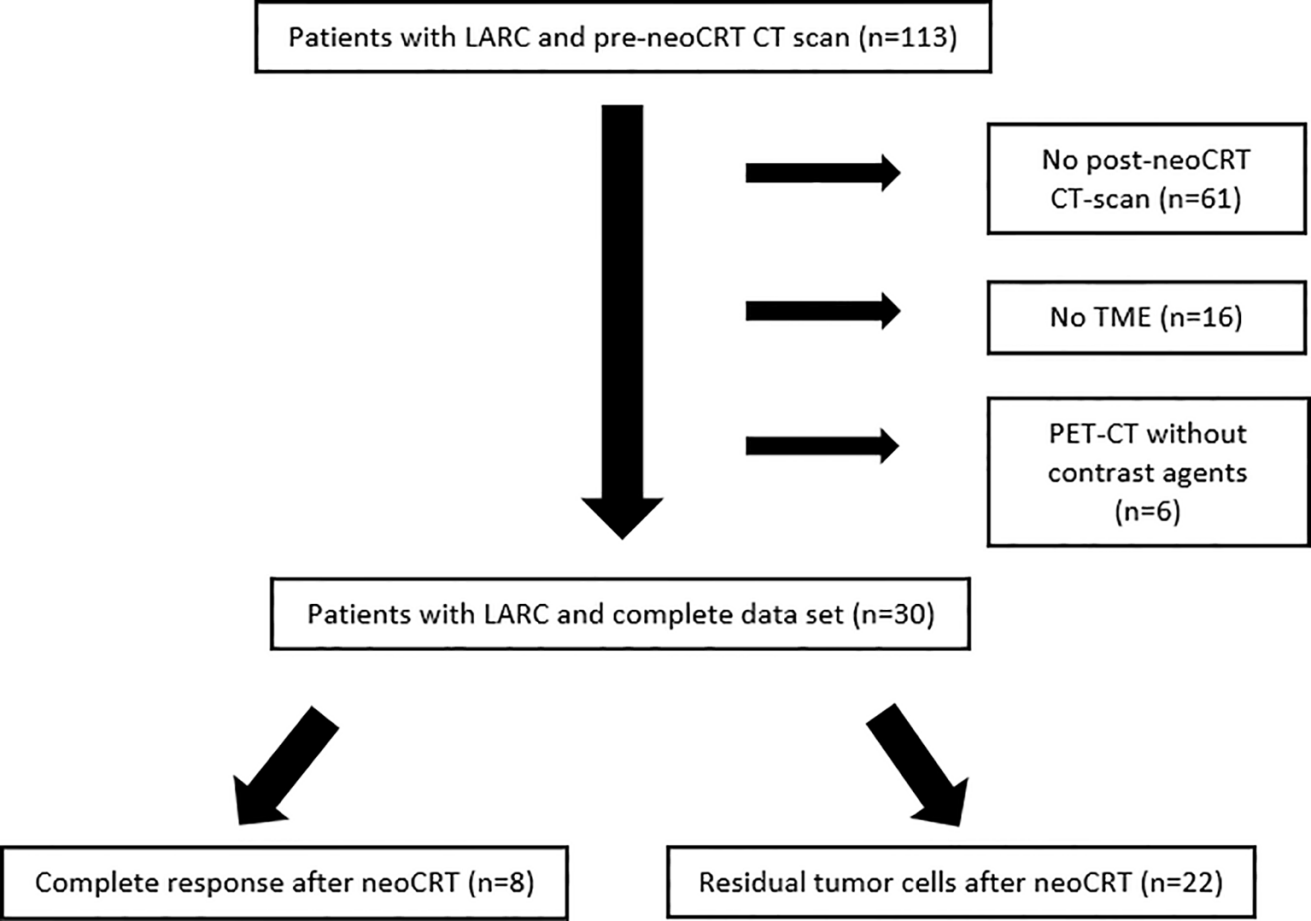
Patients with LARC and pre-neoCRT CT-scan screened for the study between 06/2012 and 04/2020. Most patients were excluded because of missing post-neoCRT CT.

We identified 113 patients with LARC which had an initial ceCT scan at the time of diagnosis. All patients underwent surgery in our institution. Of those, 83 were excluded for different reasons, mostly because of missing post neoCRT CT-scans (**Figure 2**). We retrospectively assessed pre- and post-neoCRT CT scans of 30 patients fulfilling the inclusion criteria. Most patients had received neoCRT comprising radiotherapy with 50.4 Gy (1.8 Gy daily) and 5-FU (1000 mg/m^2^, days 1 – 5) in the first and fifth week or capecitabine in equivalent doses, followed by TME (n=26). Four patients were treated in multicenter studies testing FOLFOX as a chemotherapy regimen parallel to radiotherapy (50.4 Gy). Mean timespan between ceCT-scans (pre- and post-neoCRT) CT scan was 15.4 weeks (95%-CI: 14.5 – 16.7) and mean timespan from post-neoCRT CT scan until surgery was 2.8 weeks (95%-CI: 2.1 – 3.4) Post-neoCRT MRIs were available for 25/30 patients, endosonographic diagnostics had been performed for 21/30 patients pre-RCT. Missing MRI were due to patients´ denial or contraindications e.g., pacemaker implantation. The initial pretreatment stages of the patients in MRI and EUS and pathological stages are listed in **Table S2**.

### Change of ceCT Based Densitometry in Pre- and Post-neoCRT Samples

**Figure 3:**
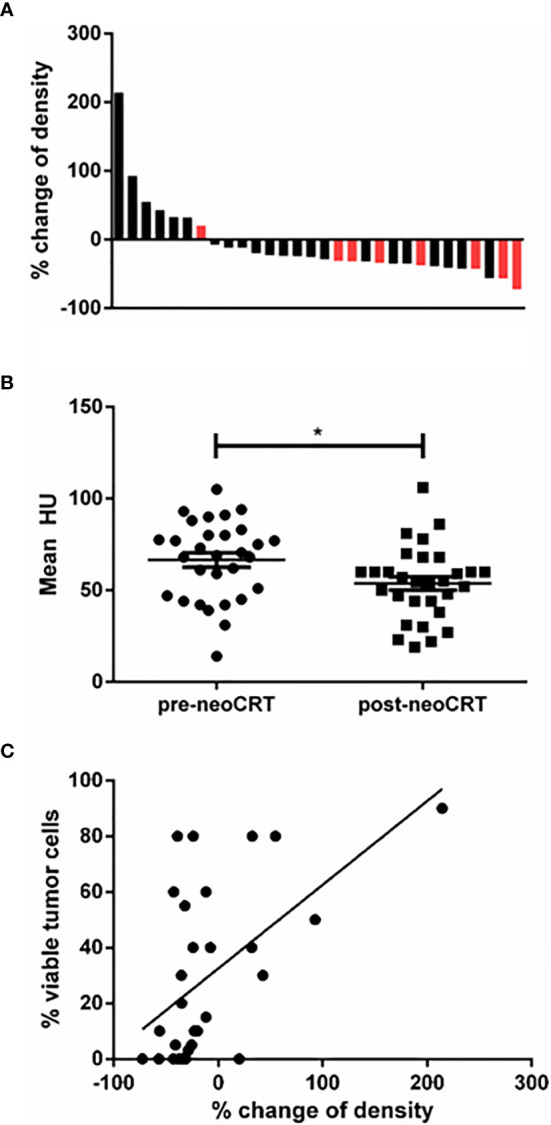
**(A)** Waterfall plot of the increase and decrease of ceCT-based density. Black: patients without pCR; red: patients with pCR. Relative change is indicated as percent of the initial mean HU measured. 8 patients had increasing density and 22 had decreasing density. Greatest decrease was 72%. **(B)** Mean HU of all tumor samples pre- and post-neoCRT (p=0.017). **(C)** Correlation between viable tumor cells in the histopathologic specimen and change of ceCT based density (r²=0.30, p=0.002). * is a symbol for significant differences.

The change of ceCT-based HU in a distinct ROI between pre- and post-neoCRT CT-scans was analyzed for each patient included. For two patients, the ROI had to be set according to visible tumor lesion in MRI, because no obvious tumor was detectable in ceCT. After neoCRT, 22 of 30 patients had a lower density of the tumor sample (73%), with the largest decrease of 72% (**Figure 3A**). Overall, the density was significantly lower following neoCRT according to the measured HU (p=0.019) (**Figure 3B**).

To analyze whether the decline of tumor density was associated with the content of residual vital tumor cells in the tumor sample, the density’s percentage of decline was correlated to vital tumor cells in the histologic sample. As depicted in **Figure 3C** we found the difference of density and the amount of residual vital tumor cells correlating (p=0.002, r^2 ^= 0.30).

### Change of ceCT-Based Densitometry in Distinct ROI Identifies pCR With High Specificity

**Figure 4:**
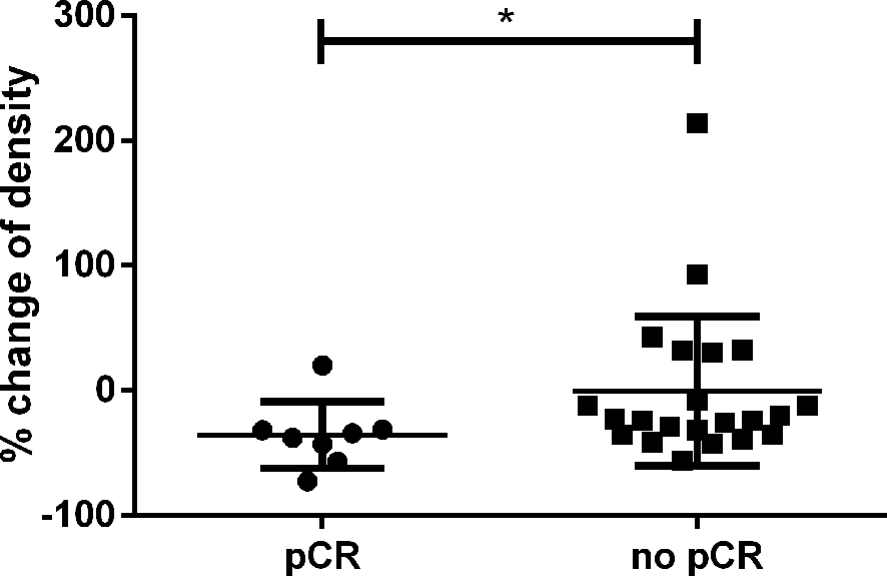
Relative change of CT-based density in percent according to the initially measured HU. Patients with pCR had a significantly greater decline than patients with residual tumor cells (p=0.030). * is a symbol for significant differences.

Next, we compared the relative decline of ceCT based density of patients with pCR (n=8) and those with vital residual tumor cells (n=22). The cut-off for pCR was set to no vital tumor cells in the histopathologic sample. We found patients with pCR to display a greater decline of density than patients with residual tumor cells (**Figure 4**), p=0.030). Receiver operating curves (ROC) analysis revealed a decline of >30% in HU as optimal cut-off to identify pCR (**Figure S1**). Of 8 patients achieving pCR, 7 had a decline above 30% in HU based densitometry, resulting in a specificity to identify pCR of 87.5%. The sensitivity to identify residual tumor cells was 68.2%, but if the tumor density did not decline greater than 30%, the probability of finding residual tumor cells in the histologic sample was very high (NPV 94%).

The absolute HU values in pretreatment ceCT based densitometry were not predictive for response (p=0.616 and AUC 0.554) (**Figure S2**). In contrast, the absolute HU values postCRT were predictive for response with p=0.03, with a lower AUC 0.75 compared to the percentage- change of density (**Figure S3**). Additionally, the absolute values in post-treatment ceCTs result in a significantly poorer specificity (**Figure S3**).

### Response Towards neoCRT Assessed by MRI and Endosonography

To compare the results of ceCT based densitometry in identifying pCR with current standard procedures, we analyzed pre-OP MRI and rectal endosonography results (**Table 1**). According to MRI, 36% of patients had a complete response (CR), 64% had a partial response (PR) and none had stable disease (SD) or progressive disease (PD). 16% of the patients did not undergo MRI. Overall, the response rate (ORR) towards neoCRT analyzed by MRI was 100%. The endosonographic ultrasound revealed a CR-rate of 19%, a PR-rate of 71% and a SD-rate of 10%, resulting in an ORR of 90%. Of all patients where results of both examinations were available (n = 18), 8 of 18 individuals had discrepant results between endosonographic examination and MRI. Only 2 patients (11%) had concordant CR in both examinations.

**Table 1:** Comparison between different imaging techniques.

Technique	pCR	Residual tumor cells	Specificity	Sensitivity	PPV	NPV	Accuracy
**Pathology**	8	22	–	–	–	–	–
	(27%)	(73%)					
**ceCT based densitometry**	14	16	88%	68%	50%	94%	73,3%
	(47%)	(53%)	(7/8)	(15/22)	(7/14)	(15/16)	(22/30)
**MRI**	9	16	50%	71%	44%	75%	64%
	(36%)	(64%)	(4/8)	(12/16)	(4/9)	(12/16)	(16/25)
**Rectal endosonography**	4	17	60%	94%	75%	88%	85,7%
	(19%)	(81%)	(3/5)	(15/16)	(3/4)	(15/17)	(18/21)

In the first row, histopathologic results are shown. Rows 2-4 indicate the results of the different imaging techniques. Column 1 + 2 show the number of patients referred as pCR or residual tumor cells, according to the respective imaging technique. Columns 3-7 show the results of the respective 2-by-2 tables.

The histopathologic examination of the tumor samples revealed that 27% (n=8), of all patients included, had pCR with no residual tumor cells. The specificity of MRI to identify pCR was 50% (4 of 8). Of 9 patients achieving CR in MRI, only 4 patients had pCR (positive predictive value (PPV) = 44%). Of 16 patients without pCR, 12 were correctly identified by MRI (negative predictive value (NPV) = 75%). The specificity of endosonographic ultrasound to identify pCR was 60% (3 of 5). Of 4 patients achieving CR in endosonographic examination, 3 had pCR (PPV = 75%). Of 17 patients not having CR assessed to endosonographic ultrasound, 15 were correctly identified (NPV = 88%). McNemar’s test showed no significant differences between ceCT based densitometry, MRI or endosonographic ultrasound (**Tables S3, S4**).

## Discussion

NeoCRT, as the standard treatment strategy, has significantly improved the rates of sphincter preservation and reduced local recurrences in LARC (5). Overall, the survival was improved for patients achieving pCR after neoCRT (5, 7). The radical surgical approach after neoCRT is increasingly questioned in patients achieving clinical complete response (cCR), since watchful waiting for these patients has shown promising results in recent clinical trials (18–20). Watch-and-wait or local surgery strategies reduce morbidity by multiple factors compared to TME (19). However, the essential premise for clinical implementation of nonsurgical treatment approaches is the precise identification of patients with cCR and assurance of high-grade concordance between cCR and pCR. Currently, there is no standard method to certainly confirm pCR. Of all patients achieving clinical CR after neoCRT, 56% had residual cancer cells in the bowel walls (13). On the other hand, 8.3% of patients who did not achieve cCR had no residual tumor cells in the histopathologic specimen (pCR) (8). Thus, additional methods to assess the treatment’s response after neoCRT are urgently needed, enabling the implementation of new treatment strategies.

In a meta-analysis performed by de Jong et al., MRI, CT, and rectal ultrasound were evaluated to predict complete response (21). The pooled estimates for CT were: sensitivity 96%, specificity 21%, PPV 86%, NPV 53%, and accuracy 83%. However, these studies analyzed tumor metrics but not ceCT based densitometry. In the present study, we found ceCT based densitometry is suitable for identification of pCR in CRC following neoCRT, with a specificity of 87.5%. Furthermore, negative predictive value to rule out pCR was 93.75%. Thus, ceCT based densitometry could improve diagnostic imaging after neoCRT and may support the implementation of new treatment approaches.

The standard response assessment after neoCRT comprises of digital-rectal examination (DRE), endoscopy, and MRI. Prediction of a clear resection margin at the mesorectal fascia is one major goal of preoperative imaging. As a major obstacle, extensive fibrosis and edema impair the diagnostic accuracy of MRI after neoCRT (22, 23). Studies evaluating the feasibility of MRI to predict complete response are heterogenous. Recent studies showed low concordance between MRI and histopathological findings (11, 22). In a meta-analysis of 16 studies, pooled estimates were sensitivity 95%, specificity 31%, PPV 83%, NPV 47%, and accuracy 75% (21). Other studies demonstrate neither diffusion-weighted magnetic resonance imaging (DWI) nor 18F-fluorodeoxy-glucose are feasible techniques to overcome the limitations of MRI in this field (24, 25). However, contemporary studies are evaluating novel MRI grading systems to enhance the prediction of tumor regression after neoCRT, with promising results and DWI is recommended by current guidelines (26–28).

As an invasive approach to identify pCR, biopsies of the primary tumor region were performed after neoCRT. However, this approach demonstrated low sensitivity of 12.9% and a poor concordance rate of 30.4% between biopsy specimens and surgical specimens (29).

The comparison of MRI and ceCT was not the primary focus of our study. Thus, conclusions drawn are limited due to low sample numbers. However, our results are in line with previous studies demonstrating limitations predicting pCR by MRI with a limited accuracy. Accordingly, only 4 of 8 patients who achieved pCR were correctly identified by MRI, indicating the known limitations of this approach (11, 13, 22). The benefit of using ceCT may result from recognizing low perfused fibrotic tumors. When compared to other studies investigating the utility of MRI for response assessment, our approach shows superior specificity and sensitivity identifying pCR (11, 13, 22). McNemar’s test to analyze the difference between the methods was performed showing no significant difference. However, using McNemar´s test in this case, methods are compared without knowing the true reference, which is the pathology of the resected tumor. Thus, the significance of the test is limited.

We consider our approach as a useful additional tool in post-neoCRT examinations. Combined MRI, endosonographic ultrasound, and ceCT-based densitometry could enhance the safety of individual treatment strategies to avoid TME. This may be of particular interest in patients not able to undergo MRI for different reasons (denial, pacemaker etc.). This is in line with the 2016 ESGAR recommendations, where a majority of the panel agreed that a multimodal approach is needed for disease staging after neoCRT, since MRI alone seems not suitable for accurate disease staging after neoCRT (28).

The discrimination between benign and malignant lesions is a major obstacle of conventional radiologic imaging, and CT-based densitometry was used in other studies to overcome this limitation. For adrenal incidentaloma, non-enhanced CT and HU of ROI can be used to discriminate benign from malignant lesions (14, 16). In colon cancer, ceCT analysis in the portal venous phase is the current standard method for initial disease staging (30). Ravanelli et al. used texture analysis of ceCTs for response prediction in patients treated with bevacizumab, with a remarkable correlation of OS and texture analysis in this subgroup of patients (31). Besides texture analysis, CT-density of lesions differed significantly between responders and non-responders in the same subgroup (31). These results indicate the different characteristics of tumor lesions responding to chemotherapy. However, measurement at a distinct point in time bares the bias of a wide-ranging lesion-density between different patients. In contrast, our approach excludes the possible bias *via* matched analysis of two CT-exams (pre- and post neoCRT).

The limitations of our study are the retrospective analysis and the small cohort size, increasing the risk of selection bias. Pre- and post-neoCRT CT-scans are, thus far, not recommended as diagnostic procedures and not performed routinely. To date, abdominal ultrasound and conventional chest imaging are suitable for disease staging. A major reason for the exclusion of most patients was the missing combination of pre- and post-neoCRT CT-scans additionally to the mandatory completion of all neoCRT cycles as well as subsequent surgery. This limits the study’s clinical validity. However, based on our preliminary results, ceCT densitometry is a promising tool to extend and enhance pre-surgery diagnostics, which encourages further research, particular in patients with no nodal involvement. Moreover, our approach is easy to perform in clinical practice, compared to radiomics. In summary, a prospective study including a larger collective is needed to validate our results.

## Data Availability Statement

The data analyzed in this study is subject to the following licenses/restrictions: patient data. Requests to access these datasets should be directed to thomas.mika@rub.de.

## Ethics Statement

The studies involving human participants were reviewed and approved by Ethik-Kommission der Medizinischen Fakultät der Ruhr-Universität Bochum. Written informed consent for participation was not required for this study in accordance with the national legislation and the institutional requirements.

## Author Contributions

MM, HH and AB collected the patient data. MM, HH, KS, MT, ME, AB and TM performed data analysis. MM, AB, RS and TM wrote the manuscript. All authors contributed to the article and approved the submitted version.

## Funding

This study was supported by a grant from de.NBI (grant number: FKZ 031 A 534A [REF]), a project of the German Federal Ministry of Education and Research (BMBF).

## Conflict of Interest

The authors declare that the research was conducted in the absence of any commercial or financial relationships that could be construed as a potential conflict of interest.
