# Supplementary material for: Computertomography-Based Prediction of Complete Response Following Neoadjuvant Chemoradiotherapy of Locally Advanced Rectal Cancer
Source: Front Oncol. 2021 May 31;11:623144. doi: 10.3389/fonc.2021.623144 (PMC8202275; doi:10.3389/fonc.2021.623144)
Supplement: Supplementary file 1 [file DataSheet_1.pdf]

# Supplemental Material

# Table S 1

# CT-scan settings

## Settings

| | arterial phase | portal-venous phase |
|---|---|---|
| tube voltage [**kV**] | 120 | 120 |
| eff. mAs [**mAs**] | <250 | <250, cave dose automatic |
| Kollimation [slices x thickness in **mm**] | 64x 0,6 or 40x0,6 | 64x0,6 40x0,6 |
| rotation time [**s**] | 0,5 | 0,5 |

## Parameter-Details

| | arterial phase | portal-venous phase |
|---|---|---|
| width [**HE**] | 350 | 350 |
| denter [**HE**] | 50 | 50 |
| length [**mm**] | 300 | 500 |
| field of View [**mm**] | T300 | 300 |
| matrix [**256,512,1024**] | 512 | 512 |
| topogram length [**mm**] | | |
| direction [**caudocranial, craniocaudal**] | cranio-caudal | |

## Contrast Agent

| | arterial phase | portal-venous phase |
|---|---|---|
| Imeron [**mg Jod/ml**] | 400 | |
| volume [**ml**] | up to 80ml depending on body weigth | |
| injection speed [**ml/s**] | 3 | |
| point of reference-ROI | Aorta abdominalis, Delay of 10 s | |
| delay [**sec.**] | bolustracking | 70 |
| duration | ca. 10 s | 15-20 s |
| bolustracking (BT) Präferenz | yes | |
| threshold reference-ROI [**HU**] | 140 | |
| NaCl-flush | yes | |
| volume NaCl [**ml**] | 40 | |
| injection speed NaCl [**ml/s**] | 3 | |
| contrast agent [**Imeron**] | Imeron 400 MCT | |

## Figure S 1

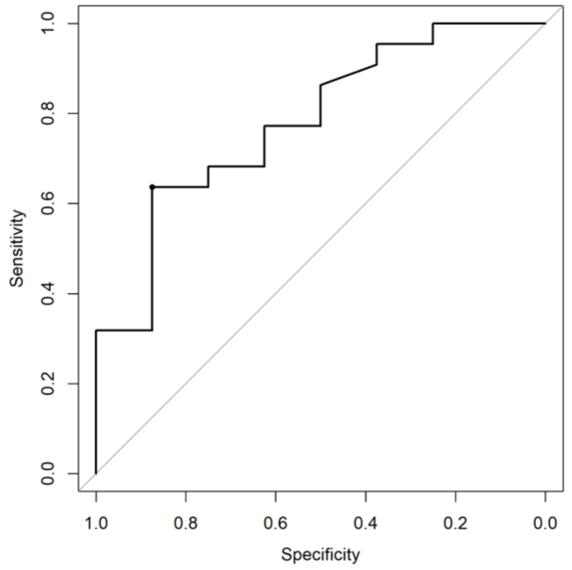


*Figure S 1* Receiver Operating Curve with a cut-off set to -30% change of ceCT based density. AUC 0.77, Sensitivity 0.875, Specificity 68.2.

## Table S 2

| ID | pretreatment MRI | pretreatment EUS | pathological stage (resectate) |
|---|---|---|---|
| BoR1 | T3N1 | T3N+ | ypT0 pN0 (0/23)L0 V0 Pn0, |
| BoR2 | T4N2 | T3N- | ypT3b pN0 (0/21) L1 V0 Pn0 |
| BoR3 | T3N2 | T3N+ | ypT2 pN0 (0/20) L0 V0 Pn0 |
| BoR4 | T3N2 | T3N+ | ypT3b pN2b (19/25) L1 V0 Pn0 |
| BoR5 | T4N2 | T4N+ | ypT3c pN0 (0/20) L0 V0 Pn0 |
| BoR6 | T2N2 | T2N- | ypT2 pN0 (0/22) L1, V0, Pn0 |
| BoR7 | T3N2 | T3N+ | ypT3b, pN2a (6/20), L1, V0, pN0 |
| BoR8 | T3N0 | stenos. | ypT3c pN0 (0/21), L0, V0, Pn0 |
| BoR9 | T3N2 | T3N+ | ypT3d Pn0 (0/21), L0, V0, P0 |
| BoR10 | T3N1 | stenos. | ypT3c pN2 (2/16) |
| BoR11 | T4N2 | T3N+ | ypT3b pN1b (5/21) L1V1Pn0 |
| BoR12 | T4N0 | T4N- | ypt4b, pN0 (0/27), L0, V0, Pn0 |
| BoR13 | T3N2 | T3N0 | ypT0 pN0 (0/22) L0 V0 Pn0 |
| BoR14 | T3 N1 | T3N+ | ypT0 pN0 (0/19) V0 L0 Pn0 R0 |
| BoR15 | T3N1 | T3N+ | ypT3c pN0 (0/23) L0, V0, Pn0 |
| BoR16 | T4N1 | T3N+ | ypT3c pN2a (6/26), |
| BoR17 | T3N0 | T3N+ | ypT0, pN0 (0/26) L0 V0 Pn0 |
| BoR18 | T3N1 | T2N+, | ypT0, pN0, L0, V0, Pn0, |
| BoR19 | not performed | T3N+. | YpT4b, ypN2b(16/44)/L1 V1, |
| BoR20 | T3N0 | T3N0 | pT2, pN0 (0/13), N0, L0, V0, |
| BoR21 | T2N1 | T2/3N0 | ypT3a, pN0 (0/27), L0, V0, Pn0 |
| BoR22 | T3N1 | uT3N+. | ypnT1A, (1/33), L0, V0, pN0 |
| BoR23 | T3N1 | uT3N+. | ypT4b, N1, L0, V0, R0 |
| BoR24 | T3N0 | T3N- | ypT2 pN1b(2/29) L0 V0 Pn0 |
| BoR25 | T3N2 | T3N+ | ypT0 pN0 (0/20) L0 V0 pN0 |
| BoR26 | T2N1 | T2N+ | ypT0, pN0 (0/11) L0, V0, Pn0 |
| BoR27 | T2N0 | T3N0 | ypT3 N1 LO V0 Pn0 |
| BoR28 | T4N0 | T4N0 | ypT3b pN0 (0/17) L1 V1 Pn0 |
| BoR29 | T3N2 | T3N0 | ypT2 pN0 (0/15) L0 V0 Pn0 |
| BoR30 | T2N1 | T2N0 | ypT0 pTN0 (0/36) L0 V0 Pn0 |

*Table S2:* The initial pretreatment stages of the patients in MRI and EUS and pathological stage.

# Figure S 2

**A**

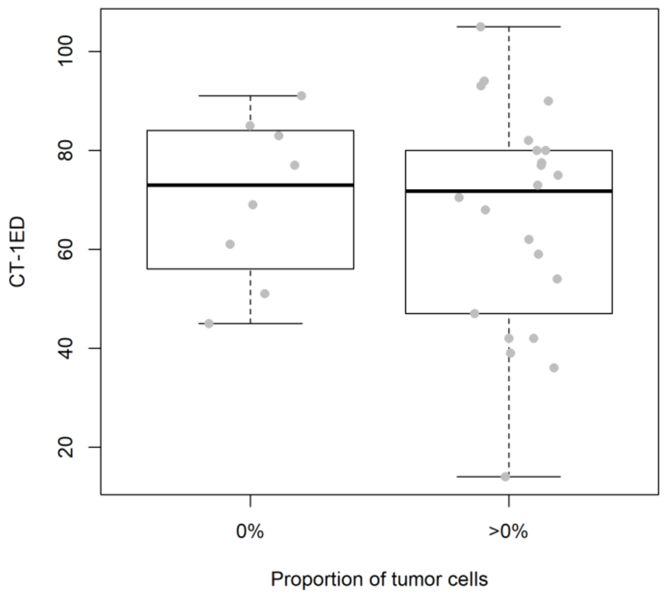


***B***

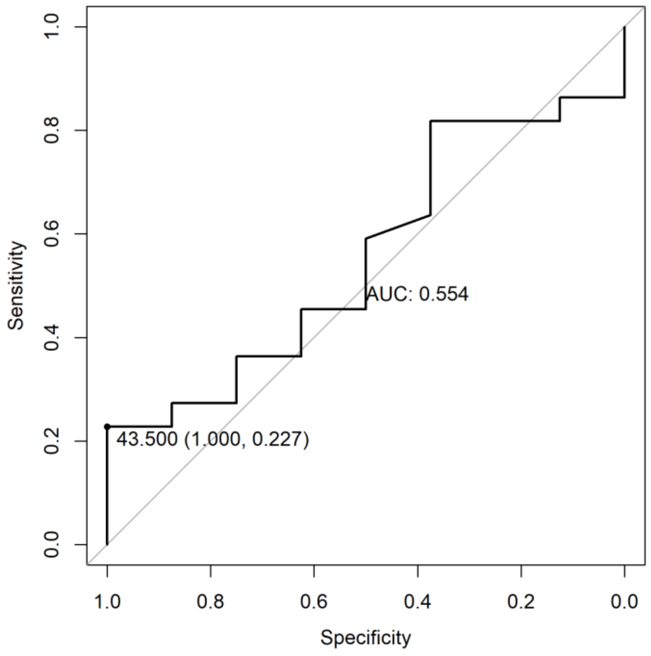


*Figure S 2* The absolute HU values in densitometry preCRT were not predictive for response (p=0.6166 and AUC 0.554)

**Figure S 3**

**A**

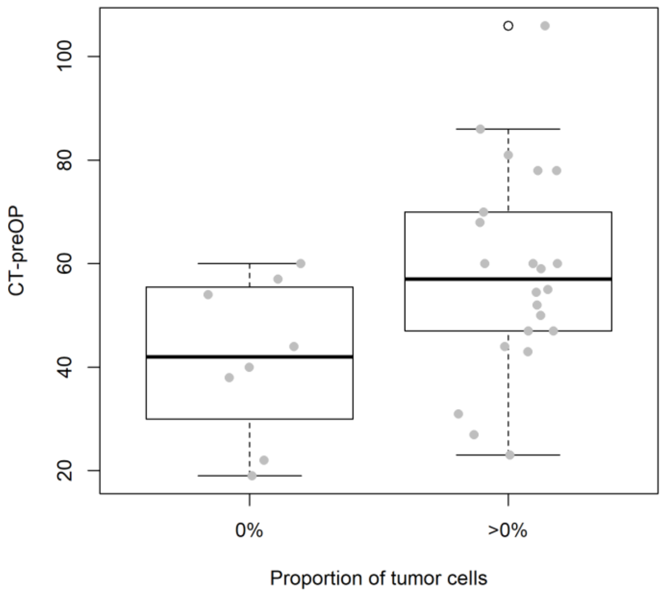


**B**

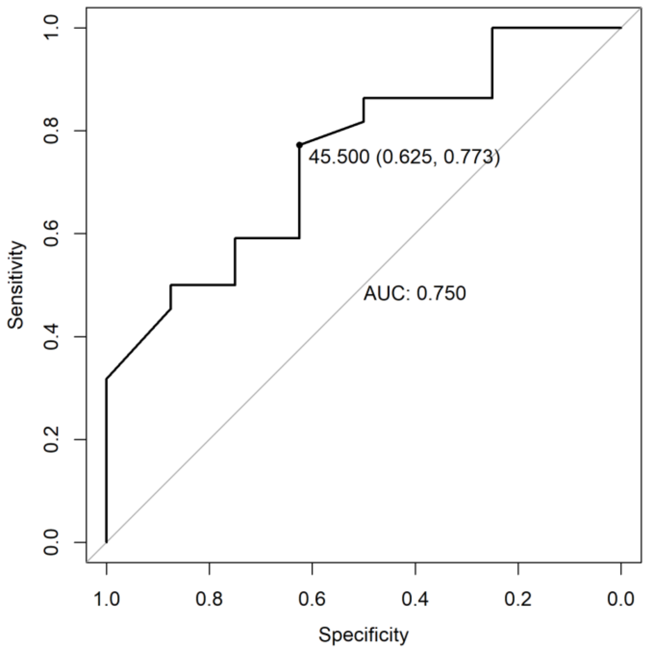


*Figure S3:* The absolute HU values in densitometry after neoCRT were predictive for response **A:** Box plot of absolute HU values after neoCRT in patients with complete response (0% residual tumor cells) and patients having residual tumor cells. **B:** ROC of absolute HU values (p=0.03 and AUC 0.75).

## Table S 3

***Comparison between ceCT based densitometry (< -30% corresponds to the complete response) and MRI, predicting therapy response***

| | results MRI | |
|---|---|---|
| %change in ceCT based densitometry | **CR** | **PR** |
| **CR (< -30)** | 6 | 8 |
| **PR (> -30)** | 3 | 8 |

*p-Value McNemar´s test: 0.227*

## Table S 4

***Comparison between ceCT based densitometry (< -30% corresponds to the complete response) and endosonographic ultrasound, predicting therapy response***

| | result endosonography | |
|---|---|---|
| %change in ceCT based densitometry | CR | PR or SD |
| CR (< -30) | 2 | 9 |
| PR (> -30) | 2 | 8 |

*p-Value McNemar´s test: 0.070*
